# Associations between serum 25-hydroxyvitamin D and bone turnover markers in a population based sample of German children

**DOI:** 10.1038/srep18138

**Published:** 2015-12-15

**Authors:** E. Thiering, I. Brüske, J. Kratzsch, L. C. Hofbauer, D. Berdel, A. von Berg, I. Lehmann, B. Hoffmann, C. P. Bauer, S. Koletzko, J. Heinrich

**Affiliations:** 1 Institute of Epidemiology I, Helmholtz Zentrum München- German Research Center for Environmental Health, Neuherberg, Germany; 2 Division of Metabolic and Nutritional Medicine, Dr. von Hauner Children’s Hospital, University of Munich Medical Center, Munich, Germany; 3 Institute of Laboratory Medicine, Clinical Chemistry and Molecular Diagnostics, University Hospital Leipzig, Leipzig, Germany; 4 Division of Endocrinology, Diabetes, and Bone Diseases, Dresden Technical University Medical Center, Dresden, Germany; 5 Research Institute, Department of Pediatrics, Marien-Hospital Wesel, Wesel, Germany; 6 Department of Environmental Immunology, Helmholtz Centre for Environmental Research — UFZ, Leipzig, Germany; 7 Medical School, the Heinrich Heine University of Düsseldorf, Düsseldorf, Germany and IUF Leibniz Research Institute for Environmental Medicine at the University of Düsseldorf, Düsseldorf, Germany; 8 Department of Pediatrics, Technical University of Munich, Munich, Germany; 9 Division of Pediatric Gastroenterology and Hepatology, Dr. von Hauner Children’s Hospital, University of Munich Medical Center, Munich, Germany; 10 Institute and Outpatient Clinic for Occupational, Social and Environmental Medicine, University Hospital Munich, Ludwig Maximilians University Munich, Germany

## Abstract

Severe vitamin D deficiency is known to cause rickets, however epidemiological studies and RCTs did not reveal conclusive associations for other parameters of bone health. In our study, we aimed to investigate the association between serum levels of 25(OH) vitamin D and bone turnover markers in a population-based sample of children. 25(OH)D, calcium (Ca), osteocalcin (OC), and β-Crosslaps (β-CTx) were measured in 2798 ten-year-old children from the German birth cohorts GINIplus and LISAplus. Linear regression was used to determine the association between bone turnover markers and 25(OH)D levels. 25(OH)D, OC, and β-CTx showed a clear seasonal variation. A 10 nmol/l increase in 25(OH)D was significantly associated with a 10.5 ng/l decrease (p < 0.001) in β-CTx after adjustment for design, sex, fasting status, time of blood drawn, BMI, growth rate, and detectable testosterone/estradiol. For OC alone no significant association with 25(OH)D was observed, whereas the β-CTx-to-OC ratio was inversely associated with 25(OH)D (−1.7% change, p < 0.001). When stratifying the analyses by serum calcium levels, associations were stronger in children with Ca levels below the median. This study in school-aged children showed a seasonal variation of 25(OH)D and the bone turnover markers OC and β-CTx. Furthermore a negative association between 25(OH)D and the bone resorption marker β-CTx was observed.

Vitamin D can be obtained by diet, however most of the body’s vitamin D is formed from 7-dehydrocholesterol within the epidermal layer of skin after exposure to UVB radiation. Vitamin D is then transported to the liver and converted to 25-hydroxyvitamin D (25(OH)D) which is the major circulating and storage form of vitamin D with a half-life of about 2 weeks^1^ and thereby reflecting the current vitamin D status. The active form calcitriol (1,25(OH)_2_D), is mainly, but not exclusively produced by 1α-hydroxylation of 25(OH)D in the kidney. The main function of calcitriolis increasing calcium calcium absorption from the gut^2^. The perception of vitamin D’s importance for bone health is well established, as severe vitamin D deficiency or very low calcium intake cause rickets - a defective mineralization of bones- in the growing child. Besides its role in intestinal calcium absorption, calcitriol may also affect bone health directly, as its receptors are expressed by osteoblasts^3^,^4^. However the relative importance of these mechanisms and their role in the growing skeleton of healthy children is unclear.

Clinical practice guidelines emphasize the importance of vitamin D in bone health. However systematic reviews of randomized controlled trials in children and adolescents^5^ as well as adults^6^ did not show significant effects on bone mineral density after vitamin D supplementation, especially in individuals with normal vitamin D levels. Therefore, we aimed to investigate the association between serum levels of 25(OH)D and bone turnover markers in a large population-based sample of children, and the effects of serum calcium levels on this association.

## Methods

The study population consists of 2,798 participants of two German birth cohorts. In both cohorts only healthy full-term neonates were recruited. The German Infant Study on the influence of Nutrition Intervention plus environmental and genetic influences on allergy (GINIplus) is a multicentre, two armed study consisting of 5,991 new-borns. One study arm is a prospective, double-blind, randomised intervention trial with hypoallergenic formulae, while the second arm is observational and does not include an intervention. The study design has been previously described in detail^7^. The Lifestyle-related factors on the Immune System and the development of Allergies in childhood (LISAplus) study consists of 3,097 healthy neonates, recruited at birth, who have a birth weight greater than 2500 g. LISAplus was designed as a population-based observational study and children have been followed up at the age of six, twelve and 18 months and two, four, six and ten years^8^. Parents of the study participants gave written informed consent. The study complied with the Ethical Principles of the World Medical Association Declaration of Helsinki and experimental protocols were approved by the regional ethics committees (Bavarian Board of Physicians, Ethics Commission of the Medical Faculty at the University of Leipzig, and Board of Physicians of North-Rhine-Westphalia).

The present analysis is restricted to 2,798 children who attended a clinical examination at age 10 years and had valid information on 25(OH)D and bone turnover markers.

### Laboratory Analyses

Blood samples, either fasting or non-fasting, were drawn at age of 10 years between November 2006 and May 2009 during all seasons and months, centrifuged after collection, and stored frozen at –80° until assayed for bone turnover markers and 25 (OH)D. Total serum 25 (OH)D concentration was determined by Roche “vitamin D total” on the fully automated Modular system (E170, Roche Diagnostics, Mannheim, Germany). The specificity is reported by the manufacturer as 25(OH)D2 = 81%; 25(OH)D3 = 98%; 1,25(OH)2D2 = 6%; 1,25(OH)2D3 = 5%; 24,25(OH)2 = 121%, and the lower limit of detection as 7.5 nmol/L. The intra-assay coefficient of variation was 2.2-6.8% for sera with levels between 20.8–173.7 nmol/L, the inter-assay coefficient of variation as provided by the manufacturer was 3.4–13.1% for levels between 20.8–173.7 nmol/L. According to the manufacturer and further validations^9^,^10^ the “vitamin D total” immunoassay shows a good comparability with LC-MS/MS.

The automated Modular system (E170, Roche) was used to measure the two bone turnover marker osteocalcin (OC) and β-isomer of the C-terminal telopeptide (β-CTx) as well as calcium (Ca), 17beta-estradiol, and 5alpha-testosterone in the same lab. Intra- and inter-assay coefficients of variation varied between 3.2% and 2.9% for concentrations of 4.1 and 21.1 nmol/l osteocalcin, 4.7% and 4.8% for concentrations of 343 and 792 ng/l CTx, and between 2.1% and 1.5% for concentrations of 2.0 and 2.9 nmol/l calcium, respectively. The analytical sensitivity was 0.087 nmol/l for testosterone and 18.4 pmol/l for estradiol. Intra- and interassay coefficients of variation were below 4.06% and 2.83% for testosterone concentrations of 6.2 and 20.2 nmol/l, respectively. For estradiol concentrations of 378 and 1941 pmol/l, intra- and interassay coefficients of variation were lower than 5.29% and 3.56%.

### Covariates

Equivalent net income was calculated by dividing the household income by the number of people in the household (the first adult weighted with factor 1, other household members at least 14 years old with factor 0.5, and all other household members with factor 0.3). Equivalent net income was then categorized into city-specific tertiles, with cutoffs 972 and 1310 Euro/month for Wesel, 1406 and 2104 Euro/month for Munich, 1086 and 1528 Euro/month for Leipzig, and 1071 and 1548 Euro/month for Bad Honnef. Maternal and paternal education level was categorized into less than 10 years, 10 years or more than 10 years of education. Height and weight were measured at age of 6 years by paediatricians during preventive health screenings and at age of 10 years during a clinical examination at the study center. Growth rate was calculated as change in age and sex standardized WHO z-score of height between age 6 and age 10 years. Physical activity was measured based on questionnaire data and categorized according to Janssen 2007^11^ as moderate and vigorous physical activity for less than 7 h per week, moderate and vigorous physical activity for more than 7 h/week, or moderate and vigorous physical activity for at least 10.5 h/week and vigorous physical activity for at least 3.5 h/week.

### Statistical analysis

All analyses were carried out using the statistical software R (version 3.1.0). Comparisons of categorical variables were performed using Fisher’s exact test in case of binary variables, and χ^2^ test for variables with more than two categories. For comparisons of normally distributed variables between groups, t-test was used.

To explore the seasonal variation of 25(OH)D, β-CTx, and OC generalized additive mixed models (gamm) as implemented in the R-packages “mgcv” were used. For month of measurement, thin plate regression splines were added to the model and the year of measurement was treated as random effect. Associations between 25(OH)D concentrations and bone turnover markers were assessed using linear regression. As adjustment variables, we tested study, city, fasting status, time of blood drawn, personal characteristics (age, sex, BMI, BMI^2^, growth rate, pubertal status, total physical activity level, time spent in front of pc/tv in summer/winter) and socioeconomic factors (parental education, single parent status and income). Only variables that showed a significant association (p < 0.05) with either OC or β-CTx in univariate models were selected for multivariable models. As the half-life time of 25(OH)D, β-CTx, and OC is very limited, we decided not to adjust for month of measurement. Regression models were additionally performed stratified by serum Ca levels and using 25(OH)D quartiles as sensitivity analyses.

## Results

**Figure 1:**
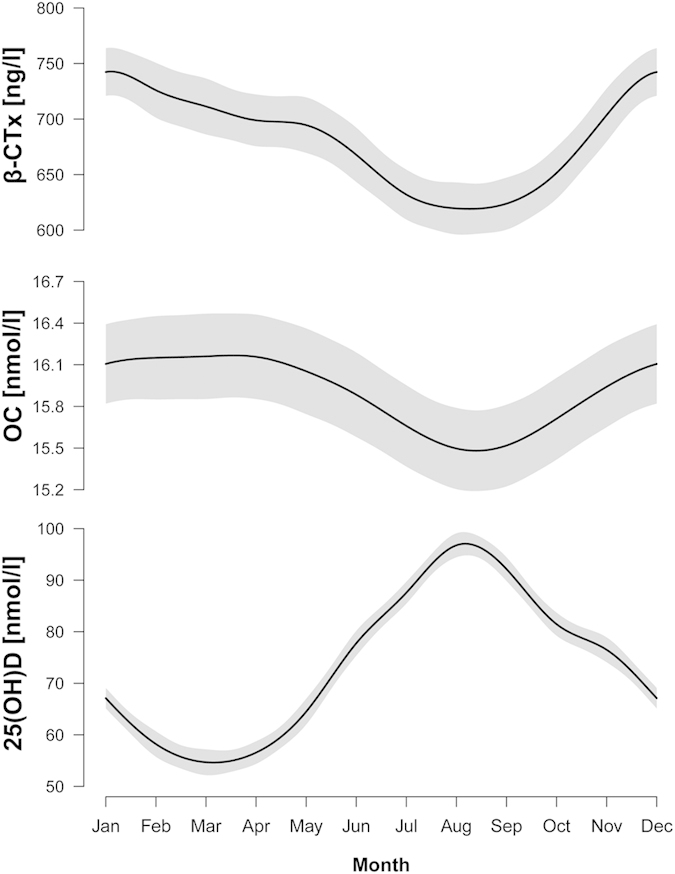
Seasonal mean estimated using generalized additive modeling adjusted for BMI, age, fasting status, time of blood drawn. Point wise confidence intervals shaded in grey.

The numbers of blood samples taken per month were: January (161), February (220), March (216), April (283), May (212), June (235), July (304), August (241), September (247), October (264), November (224), and December (191). There was a clear seasonal dependency within the measurements β-CTx, OC, and 25(OH)D (Fig. 1). 25(OH)D ranged from 21.8 nmol/l to 142.4 nmol/l with a mean of 63.1 nmol/l in the winter months from November until April, while during the summer months values from 23.2 nmol/l to 224.6 nmol/l with a mean of 83.9 nmol/l were observed. For all three parameters, a significant (p < 0.001) non-linear effect of months of measurement was seen. The adjusted R^2^ was 32.4% for 25(OH)D, 34.2% for β-CTx and 10.1% for OC.

Children had a mean age of 10.2 and 51.2% were male (Table 1). Estradiol was detectable in 72.3% of girls, whereas testosterone was only detectable in 27.7% of the boys (p < 0.001). The two bone turnover markers showed a significant positive correlation (Pearson’s ρ = 0.60, p < 0.001).

Boys had a higher physical activity level (p < 0.001), higher 25(OH)D concentrations (p = 0.009), lower OC concentrations (p < 0.001) and lower β-CTx concentrations (p = 0.009), but there was no significant difference in BMI between girls and boys (p = 0.926). Children with detectable estradiol/testosterone showed lower 25(OH)D concentrations (p < 0.001) and an increase in bone turnover markers (p < 0.0001).

A 10 nmol/l increase in 25(OH)D was associated with a significant decrease of −10.5ng/l (95%-CI: −14.9,−6.2; p < 0.001) in the bone resorption marker β-CTx when controlling for city, study, sex, fasting status BMI, BMI^2^, growth rate, time of blood drawn, and detectable sex hormones (Table 2). For the bone formation marker OC a non-significant increase of 0.03 nmol/l (95%-CI: -0.06,0.09; p = 0.795) was observed resulting in a −1.7% (95%-CI: −2.3, −1.2, p < 0.001) decrease of the β-CTx to OC ratio. Additional adjustment for maternal and paternal education and physical activity only marginally changed the association results. Fasting status, growth rate and gender had the largest effect sizes among the covariates for OC, and fasting status, growth rate, study, and city for β-CTx, but crude models (results not shown) yielded the similar results. There was no effect modification of gender in any of the models (p for interaction between gender and 25(OH)D >0.05 in all models).

When stratifying the results for serum calcium levels (median or higher versus lower than median) the association between bone turnover markers and 25(OH)D was stronger in children with calcium concentrations below the median. The formal test for an interaction between serum calcium and bone turnover marker yielded a p-value of 0.03 for β-CTx and p = 0.001 for the β-CTx to OC ratio.

In sensitivity analyses, we restricted the study population to fasting subjects, and observed slightly increased effect estimates. Also further adjustment of the models for parental income, single parent status, age, and pubertal status obtained from questionnaires did not lead to substantial differences in effect estimates. When using quartiles of 25(OH)D levels in the analysis, we observed significantly decreased β-CTx concentrations for the 2^nd^–4^th^ quartile (medium to high 25(OH)D) compared with the first quartile (low 25(OH)D) (2^nd^ quartile: β = −43.4, p = 0.006, 3^rd^ quartile: β = −69.2, p < 0.001, 4^th^ quartile: β = −67.9, p < 0.001) and no effect on OC (2^nd^ quartile: β = −0.08, p = 0.775, 3^rd^ quartile: β = 0.03, p = 0.918, 4^th^ quartile: β = 0.03, p = 0.917).

## Discussion

Our study showed a clear seasonal variation of bone turnover markers and a negative association between serum 25(OH)D and β-CTx concentrations in a large population-based sample of school-aged children, with an effect modification of serum calcium levels.

OC is solely secreted by osteoblasts and a marker for bone formation. β-CTx is a marker that is specific for bone resorption and shows a circadian rhythm. Both markers are increased during periods of rapid bone turnover such as during growth in childhood and adolescence^12^ and typically show a positive association with growth rate^13^,^14^.

Most studies on bone health have focused mainly on the elderly or post-menopausal woman owing to their greater risk of osteoporosis^15^,^16^.

Smaller observational studies investigating the association between serum levels of 25(OH)D and bone turnover markers have been performed in healthy adolescents. Jones *et al.*^17^ showed a significant association between bone turnover markers in 136 Tasmanian boys and Viljakainen *et al.*^18^ in 196 Finnish girls, whereas in 172 10–17 years old students from schools in Beirut no association was seen in samples collected during November and December^19^. A study in 138 6–12 year old youth living in Pittsburgh, Pennsylvania) who were examined once during summer (June-September) and once during winter (December-March) showed higher 25(OH)D concentrations in summer accompanied by lower CTx levels^20^. However the study did not reveal any significant association neither between 25(OH)D and CTx nor with OC. In 196 Swiss adolescents aged 11–16 years, also no association between bone turnover and 25(OH)D was observed^21^. A further study was conducted in 301 Chinese adolescent girls and revealed significantly reduced BMC and higher concentrations for bone-specific alkaline phosphatase (BAP) in vitamin D deficient individuals, but no differences were found in OC^22^.

Baseline concentrations of the calcitriol were associated with an 0.51 ng/ml increase in OC one year later in a prospective study in 178 healthy female adolescents with a mean age of 11.7 (SD = 2.3) years^23^.

Cashman *et al.*^24^ investigated the association between 25(OH)D levels and BMD measured by dual-energy X-ray absorptiometry on the forearm and heel in boys and girls of two age groups (12 years and 15 years). A significantly higher forearm BMD was observed for girls in the highest 25(OH)D tertile compared to the lowest tertile, but neither for heel BMD nor for boys any association was observed. 25(OH)D concentrations in the highest tertile were additionally associated with lower OC concentrations compared to the lowest tertile in 12 and 15-year old boys^24^,^25^. There was no association between 25(OH)D and CTx in any sex or age group.

Bone turnover markers (serum BAP, OC, urine Ntx) were also not influenced by serum 25(OH)D levels in 302 pubertal healthy black and white children enrolled during winter (October-January), neither at baseline nor after 12 weeks of supplementation with vitamin D3^26^. A longer supplementation of 12 months vitamin D3 did also not show any association with bone turnover markers (serum OC, urine Pyr, Dpyr) in 221 girls aged 11-12 years^27^.

Thus, results of previous observational studies in children and adolescents were inconclusive, with some showing effects of vitamin D concentrations on either bone formation or bone resorption^17^,^18^,^22^,^23^,^24^,^25^, while others did not^19^,^20^,^21^,^26^,^27^. Taken together with the notion of a lacking effect of vitamin D supplementation on bone mineral density in clinical trials^5^ there remains uncertainty about the presence and direction of an association between vitamin D status and markers of bone turnover in children and adolescents. There are several possible reasons why trials did not detect significant associations. The meta-analysis of Winzenberg *et al.*^5^ revealed a trend towards larger effects in studies of participants with low baseline 25(OH)D levels, therefore high baseline 25(OH)D concentrations might have prevented any additional effect of vitamin D supplementation. Also the dosage of supplementation in some of the trials (mostly 200 IU vitamin D) was likely too low to provoke a detectable effect. In adults, vitamin D is often supplemented together with calcium, which makes it difficult to interpret isolated effects of vitamin D.

Here, we observed lower concentrations of β-CTx in children with higher circulating vitamin D concentrations and a decreased β-CTx to OC ratio. The assumed pathophysiological basis of this association is that low 25(OH)D levels can be considered as a marker for low calcitriol levels which lead to a lower intestinal calcium absorption. To compensate for this, parathyroid hormone (PTH) is released to increase the circulating Ca levels by bone resorption and demineralization. In our study, the association of 25(OH)D and bone turnover marker was stronger in children with calcium levels below the median, also indicating that these mechanisms may be operative to prevent low calcium serum levels. In previous studies, Ca was not considered as a potential effect modifier. Some studies accounted only for dietary Ca intake^18^,^19^,^20^,^21^,^23^,^24^,^25^, others in which Ca was measured in serum treated it as outcome^22^,^27^ no study investigated the effects of vitamin D status on bone turnover marker in relation to serum Ca status.

**Figure 2:**
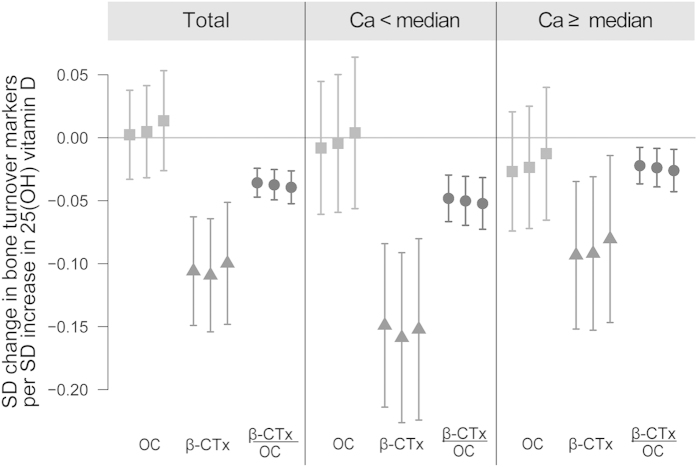
Results of linear regression on standardized values. Description: Model 1: city, study, sex, fasting status, time of blood drawn; Model 2: Model 1 + BMI, BMI2, growth rate, detectable testosterone/estradiol; Model 3: Model 2 + maternal and paternal education, center specific income tertiles, single parent status) and physical activity.

In general, the effect sizes in our study were low (about −0.10 SD decrease in β-CTx per SD increase in 25 (OH) D, and about −0.04 SD decrease in β-CTx to OC ratio, see Fig. 2. We hypothesize that the lack of power might be a plausible reason why smaller studies often did not find any statistically significant association between bone turnover markers and vitamin D status, especially in population without vitamin D deficiency and with sufficient Ca supply.

### Limitations and strength

The main strength of the present study is that it was conducted in a large population-based sample of children which allows detection of small effect sizes. Furthermore, detailed information on personal characteristics has been prospectively collected. Therefore adjustment for many covariates and potential confounders was possible.

It is challenging to measure vitamin D status as the obtained concentrations vary with the methods applied^28^. In GINIplus and LISAplus the measured 25(OH)D levels were higher than reported for this age group in the KIGGS study^29^ and NHANES^30^. Therefore, we used in our analyses 25(OH)D concentrations as continuous variable as well as in quartiles and avoided interpretation in terms of vitamin D sufficiency. To better investigate the pathophysiological basis for the observed association, an additional measurement of PTH and receptor activator of nuclear factor kappa-B ligand (RANKL) would be required. Therefore, the unavailability of these measures has to be considered as a major limitation of this manuscript. Especially as serum calcium concentration is a very poor marker for the determination of calcium status. Furthermore calcium was not albumin adjusted and thus might not reflect the true biologic effect. Furthermore, as the design of the study was observational and cross-sectional, it cannot establish a causal link between 25(OH)D levels and bone turnover.

## Conclusion

This study showed associations between a marker of bone turnover and 25(OH)D which are dependent on serum calcium level in healthy school-aged children. Higher levels of 25(OH)D were associated with slightly decreased β-CTx, and a decreased β-CTx to OC ratio. Nevertheless, clinical relevance of the findings is limited due to small effect sizes in this population with comparably high 25(OH)D levels.

## Additional Information

**How to cite this article**: Thiering, E. *et al.* Associations between serum 25-hydroxyvitamin D and bone turnover markers in a population based sample of German children. *Sci. Rep.* **5**, 18138; doi: 10.1038/srep18138 (2015).

## Figures and Tables

**Table 1:** Study population characteristics.

	All participants		Girls	Boys
Study and centre,%				
GINI Munich	36.7	1027/2798	37.6	35.8
GINI Wesel	25.7	718/2798	26.6	24.7
LISA Munich	18.8	526/2798	17.6	20.0
LISA Wesel	3.7	104/2798	3.5	3.9
LISA Leipzig	9.9	276/2798	9.4	10.3
LISA Bad Honnef	5.3	147/2798	5.2	5.3
Age [years], mean (sd)	10.2 (0.2)		10.2 (0.2)	10.2 (0.2)
Sex				
male	51.2	1432/2798	0.0	100.0
BMI [kg/m^2^]	17.4 (2.5)		17.4 (2.5)	17.4 (2.5)
Growth rate^a^	0.18 (0.50)		0.10 (0.53)	0.26 (0.45)
Detectable testosterone/estradiol^b^, %	50.0	1370/2741	74.0	27.1
Fasting status at blood drawn, %	17.4	487/2798	18.7	16.1
Time of examination,%				
before 11am	28.0	739/2641	28.8	27.2
11am–2pm	12.0	318/2641	12.9	11.3
after 2 pm	60.0	1584/2641	58.4	61.5
Physical activity,%				
moderate or vigorous physical activity <7h	27.0	633/2346	32.4	22.0
moderate or vigorous physical activity ≥7h	37.6	883/2346	39.6	35.8
moderate or vigorous physical activity ≥10.5h and vigorous physical activity ≥ 3.5 h	35.4	830/2346	27.9	42.2
Maternal education,%				
Low (<10 y)	9.5	265/2784	8.8	10.2
medium (10 y)	38.3	1065/2784	39.1	37.5
High (>10 y)	52.2	1454/2784	52.2	52.3
Paternal education, %				
Low (<10 y)	18.4	505/2747	18.0	18.7
medium (10 y)	22.6	622/2747	22.4	22.9
High (>10 y)	59.0	1620/2747	59.6	58.4
Center specific income tertiles,%				
1^st^ tertile	32.6	836/2568	31.5	33.5
2^nd^ tertile	30.3	778/2568	31.3	29.3
3^rd^ tertile	37.1	954/2568	37.2	37.1
25 (OH) D [nmol/l], mean (sd)	74.3 (25.2)		73.0 (24.6)	75.5 (25.7)
OC [nmol/l], mean (sd)	15.9 (5.3)		16.9 (5.8)	15.0 (4.7)
β-CTx [ng/l], mean (sd)	679.4 (333.4)		696.3 (351.7)	663.3 (314.9)
β-CTx/OC [ng/nmol], geometric mean (sd)	39.7 (1.5)		38.4 (1.5)	41.1 (1.5)

^a^defined as height z-score 10 years minus height z-score 6 years.

^b^0.09nmol/l for testosterone, 0.0184 nmol/l for estradiol.

**Table 2:** Results of linear regression between bone metabolism marker and 25(OH) D in different sets of adjustment.

	All			low calcium (<median)			high calcium (≥median)		
	effect^a^	(CI)	p	effect^a^	(CI)	p	effect^a^	(CI)	p
Basic model^b^									
OC	0.01	(−0.07, 0.08)	0.896	−0.02	(−0.12, 0.10)	0.765	−0.06	(−0.16, 0.04)	0.268
β-CTx	**−10.2**	**(−14.4, –6.1)**	**<0.001**	**−14.4**	**(−20.6, −8.1)**	**<0.001**	−**9.0**	(−**14.7, −3.4)**	**0.002**
β-CTx to OC ratio	−**1.7%**	(−**2.2, −1.1)**	**<0.001**	−**2.2%**	(−**3.1, −1.4)**	**<0.001**	−**1.0%**	(−**1.7, −0.4)**	**0.003**
Extended model^c^									
OC	0.01	(−0.07, 0.09)	0.795	−0.01	(−0.13, 0.11)	0.872	−0.05	(−0.15, 0.05)	0.343
β-CTx	−**10.5**	(−**14.9, –6.2)**	**<0.001**	−**15.3**	(−**21.8, –8.8)**	**<0.001**	−**8.9**	(−**14. 8, −3.0)**	**0.003**
β-CTx to OC ratio	−**1.7%**	(−**2.3, –1.2)**	**<0.001**	−**2.3%**	(−**3.2, −1.4)**	**<0.001**	**−1.1%**	**(−1.8, −0.4)**	**0.002**
Full model^d^									
OC	0.03	(−0.06, 0.11)	0.504	0.01	(−0.12, 0.14)	0.899	−0.03	(−0.14, 0.09)	0.636
β-CTx	**−9.6**	**(−14.3, −45.0)**	**<0.001**	**−14.7**	**(−21.6, –7.7)**	**<0.001**	**−7.8**	**(−14.2, –1.4)**	**0.018**
β-CTx to OC ratio	**−2.4%**	**(−2.4, −1.2)**	**<0.001**	**−2.4%**	**(−3.4, –1.5)**	**<0.001**	**−1.2%**	**(−2.0, –0.4)**	**0.003**

^a^effect: beta for OC and β-CTx, %change for β-CTx to OC ratio.

^b^Basic model: adjusted for city, study, sex, fasting status, time of blood drawn.

^c^Extended model: basic model adjustment plus BMI, BMI^2^, growth rate, detectable testosterone/estradiol.

^d^Full model: extended model adjustment plus maternal and paternal education and physical activity.
